# Supplementary material for: Efficacy and Safety of Mesenchymal Stem/Stromal Cell Therapy for Inflammatory Bowel Diseases: An Up-to-Date Systematic Review
Source: Biomolecules. 2021 Jan 11;11(1):82. doi: 10.3390/biom11010082 (PMC7827559; doi:10.3390/biom11010082)
Supplement: Supplementary file 1 [file biomolecules-11-00082-s001.pdf]

**Supplementary Table S1. PubMed Search Strategy**			
**No.**	**Search terms (PubMed)**	**Search Details (PubMed)**	**Number of records retrieved**
**1**	Stem Cell AND Inflammatory Bowel Disease	("stem cells"[MeSH Terms] OR ("stem"[All Fields] AND "cells"[All Fields]) OR "stem cells"[All Fields] OR ("stem"[All Fields] AND "cell"[All Fields]) OR "stem cell"[All Fields]) AND ("inflammatory bowel diseases"[MeSH Terms] OR ("inflammatory"[All Fields] AND "bowel"[All Fields] AND "diseases"[All Fields]) OR "inflammatory bowel diseases"[All Fields] OR ("inflammatory"[All Fields] AND "bowel"[All Fields] AND "disease"[All Fields]) OR "inflammatory bowel disease"[All Fields])	774
**2**	Stem Cell AND Ulcerative colitis	("stem cells"[MeSH Terms] OR ("stem"[All Fields] AND "cells"[All Fields]) OR "stem cells"[All Fields] OR ("stem"[All Fields] AND "cell"[All Fields]) OR "stem cell"[All Fields]) AND ("colitis, ulcerative"[MeSH Terms] OR ("colitis"[All Fields] AND "ulcerative"[All Fields]) OR "ulcerative colitis"[All Fields] OR ("ulcerative"[All Fields] AND "colitis"[All Fields]))	190
**3**	Stem Cell AND Crohn’s	("stem cells"[MeSH Terms] OR ("stem"[All Fields] AND "cells"[All Fields]) OR "stem cells"[All Fields] OR ("stem"[All Fields] AND "cell"[All Fields]) OR "stem cell"[All Fields]) AND ("crohn"[All Fields] OR "crohn s"[All Fields] OR "crohns"[All Fields])	392
**4**	Stem Cell AND Ileocolitis	("stem cells"[MeSH Terms] OR ("stem"[All Fields] AND "cells"[All Fields]) OR "stem cells"[All Fields] OR ("stem"[All Fields] AND "cell"[All Fields]) OR "stem cell"[All Fields]) AND ("crohn disease"[MeSH Terms] OR ("crohn"[All Fields] AND "disease"[All Fields]) OR "crohn disease"[All Fields] OR "ileocolitis"[All Fields] OR "ileocolic"[All Fields])	373
**5**	Stem Cell AND Ileitis	("stem cells"[MeSH Terms] OR ("stem"[All Fields] AND "cells"[All Fields]) OR "stem cells"[All Fields] OR ("stem"[All Fields] AND "cell"[All	7

		Fields]) OR "stem cell"[All Fields]) AND ("ileitis"[MeSH Terms] OR "ileitis"[All Fields])	
**6**	Stem Cell AND Colitis	("stem cells"[MeSH Terms] OR ("stem"[All Fields] AND "cells"[All Fields]) OR "stem cells"[All Fields] OR ("stem"[All Fields] AND "cell"[All Fields]) OR "stem cell"[All Fields]) AND ("colitis"[MeSH Terms] OR "colitis"[All Fields] OR "colitides"[All Fields])	614
**7**	Stem Cell AND Enteritis	("stem cells"[MeSH Terms] OR ("stem"[All Fields] AND "cells"[All Fields]) OR "stem cells"[All Fields] OR ("stem"[All Fields] AND "cell"[All Fields]) OR "stem cell"[All Fields]) AND ("enteric"[All Fields] OR "enterically"[All Fields] OR "enterics"[All Fields] OR "enteritis"[MeSH Terms] OR "enteritis"[All Fields] OR "enteritides"[All Fields])	372
**8**	Stem Cell AND Perianal	("stem cells"[MeSH Terms] OR ("stem"[All Fields] AND "cells"[All Fields]) OR "stem cells"[All Fields] OR ("stem"[All Fields] AND "cell"[All Fields]) OR "stem cell"[All Fields]) AND ("perianal"[All Fields] OR "perianally"[All Fields])	114
**9**	Stem Cell AND Fistula	("stem cells"[MeSH Terms] OR ("stem"[All Fields] AND "cells"[All Fields]) OR "stem cells"[All Fields] OR ("stem"[All Fields] AND "cell"[All Fields]) OR "stem cell"[All Fields]) AND ("fistula"[MeSH Terms] OR "fistula"[All Fields] OR "fistulas"[All Fields] OR "fistula s"[All Fields] OR "fistulae"[All Fields] OR "fistulaes"[All Fields])	213
**10**	Mesenchymal AND Inflammatory Bowel Disease	("mesenchym"[All Fields] OR "mesenchymal"[All Fields] OR "mesenchymalized"[All Fields] OR "mesenchymally"[All Fields] OR "mesenchymals"[All Fields] OR "mesenchymes"[All Fields] OR "mesoderm"[MeSH Terms] OR "mesoderm"[All Fields] OR "mesenchyme"[All Fields]) AND ("inflammatory bowel diseases"[MeSH Terms] OR ("inflammatory"[All Fields] AND "bowel"[All Fields] AND "diseases"[All Fields]) OR "inflammatory bowel diseases"[All Fields] OR ("inflammatory"[All Fields] AND "bowel"[All Fields] AND "disease"[All Fields]) OR "inflammatory bowel disease"[All Fields])	364
**11**	Mesenchymal AND Ulcerative colitis	("mesenchym"[All Fields] OR "mesenchymal"[All Fields] OR "mesenchymalized"[All Fields] OR "mesenchymally"[All Fields] OR "mesenchymals"[All Fields] OR "mesenchymes"[All	82

		Fields] OR "mesoderm"[MeSH Terms] OR "mesoderm"[All Fields] OR "mesenchyme"[All Fields]) AND ("colitis, ulcerative"[MeSH Terms] OR ("colitis"[All Fields] AND "ulcerative"[All Fields]) OR "ulcerative colitis"[All Fields] OR ("ulcerative"[All Fields] AND "colitis"[All Fields]))	
**12**	Mesenchymal AND Crohn's	("mesenchym"[All Fields] OR "mesenchymal"[All Fields] OR "mesenchymalized"[All Fields] OR "mesenchymally"[All Fields] OR "mesenchymals"[All Fields] OR "mesenchymes"[All Fields] OR "mesoderm"[MeSH Terms] OR "mesoderm"[All Fields] OR "mesenchyme"[All Fields]) AND ("crohn"[All Fields] OR "crohn s"[All Fields] OR "crohns"[All Fields])	213
**13**	Mesenchymal AND Ileocolitis	("mesenchym"[All Fields] OR "mesenchymal"[All Fields] OR "mesenchymalized"[All Fields] OR "mesenchymally"[All Fields] OR "mesenchymals"[All Fields] OR "mesenchymes"[All Fields] OR "mesoderm"[MeSH Terms] OR "mesoderm"[All Fields] OR "mesenchyme"[All Fields]) AND ("crohn disease"[MeSH Terms] OR ("crohn"[All Fields] AND "disease"[All Fields]) OR "crohn disease"[All Fields] OR "ileocolitis"[All Fields] OR "ileocolic"[All Fields])	207
14	Mesenchymal AND Ileitis	("mesenchym"[All Fields] OR "mesenchymal"[All Fields] OR "mesenchymalized"[All Fields] OR "mesenchymally"[All Fields] OR "mesenchymals"[All Fields] OR "mesenchymes"[All Fields] OR "mesoderm"[MeSH Terms] OR "mesoderm"[All Fields] OR "mesenchyme"[All Fields]) AND ("ileitis"[MeSH Terms] OR "ileitis"[All Fields])	0
15	Mesenchymal AND Colitis	("mesenchym"[All Fields] OR "mesenchymal"[All Fields] OR "mesenchymalized"[All Fields] OR "mesenchymally"[All Fields] OR "mesenchymals"[All Fields] OR "mesenchymes"[All Fields] OR "mesoderm"[MeSH Terms] OR "mesoderm"[All Fields] OR "mesenchyme"[All Fields]) AND ("colitis"[MeSH Terms] OR "colitis"[All Fields] OR "colitides"[All Fields])	266
16	Mesenchymal AND Enteritis	("mesenchym"[All Fields] OR "mesenchymal"[All Fields] OR "mesenchymalized"[All Fields] OR "mesenchymally"[All Fields] OR "mesenchymals"[All Fields] OR "mesenchymes"[All Fields] OR "mesoderm"[MeSH Terms] OR "mesoderm"[All Fields] OR "mesenchyme"[All	100

		Fields]) AND ("enteric"[All Fields] OR "enterically"[All Fields] OR "enterics"[All Fields] OR "enteritis"[MeSH Terms] OR "enteritis"[All Fields] OR "enteritides"[All Fields])	
17	Mesenchymal AND Perianal	("mesenchym"[All Fields] OR "mesenchymal"[All Fields] OR "mesenchymalized"[All Fields] OR "mesenchymally"[All Fields] OR "mesenchymals"[All Fields] OR "mesenchymes"[All Fields] OR "mesoderm"[MeSH Terms] OR "mesoderm"[All Fields] OR "mesenchyme"[All Fields]) AND ("perianal"[All Fields] OR "perianally"[All Fields])	72
18	Mesenchymal AND Fistula	("mesenchym"[All Fields] OR "mesenchymal"[All Fields] OR "mesenchymalized"[All Fields] OR "mesenchymally"[All Fields] OR "mesenchymals"[All Fields] OR "mesenchymes"[All Fields] OR "mesoderm"[MeSH Terms] OR "mesoderm"[All Fields] OR "mesenchyme"[All Fields]) AND ("fistula"[MeSH Terms] OR "fistula"[All Fields] OR "fistulas"[All Fields] OR "fistula s"[All Fields] OR "fistulae"[All Fields] OR "fistulaes"[All Fields])	139
